# Development and validation of the Gastrointestinal Symptom Severity Scale in Spanish children and adolescents

**DOI:** 10.1007/s00431-024-05504-8

**Published:** 2024-03-25

**Authors:** Néstor Montoro-Pérez, Agustín Ernesto Martínez-González, Lidia Infante-Cañete, María de los Ángeles Martínez-González, Silvia Hidalgo-Berutich, Pedro Andreo-Martínez

**Affiliations:** 1 Department of Nursing, Faculty of Health Sciences, Person-Centred Care and Health Outcomes Innovation Group, University of Alicante, San Vicente del Raspeig, Spain; 2 GREIACC Research Group, La Fe Health Research Institute, Valencia, Spain; 3 Department of Developmental Psychology and Didactics, University of Alicante, PO 99, 03080 Alicante, Spain; 4 Department of Developmental and Educational Psychology, Faculty of Psychology, University of Malaga, Malaga, Spain; 5 Ministry of Education, Vocational Training and Employment, Murcia, Spain; 6 Department of Agricultural Chemistry, Faculty of Chemistry, University of Murcia, Murcia, Spain

**Keywords:** Functional gastrointestinal disorders, Gastrointestinal symptoms, Constipation, Pain, Adolescents, Children

## Abstract

Functional gastrointestinal disorders (FGIDs) are characterized by chronic or recurrent gastrointestinal symptoms (GS) that are not explained by structural or biochemical abnormalities. FGIDs are related to lower quality of life, increased demands on medical resources, and greater somatization and emotional instability. Furthermore, GS appears to be an indicator of dysbiosis in gut-microbiota, affecting the gut-microbiota-brain relationship. To develop and evaluate the psychometric properties of a new instrument called the Gastrointestinal Symptom Severity Scale (GSSS) using a web-based survey in a sample of neurotypical children and adolescents from Spain. Instrument development and validation processes were applied to the GSSS following its administration as part of an online survey. The sample included 1242 neurotypical children and adolescents. The mean age of participants was 13.95 years, with a standard deviation of 1.37 years. Overall, 13.8% suffered infectious diarrhea, 12.6% suffered abdominal pain, 5% suffered dyspepsia and 2.6% suffered gastro-esophageal reflux. A single-factor model produced good fit indices. Furthermore, internal consistency and test-retest reliability outcomes were acceptable. The GSSS was found to have acceptable metric measurement invariance. Significant correlations with other instruments were produced and were of expected direction and magnitude, confirming scale validity for hypothesis testing.

*    Conclusions*: The GSSS shows promising psychometric properties for assessing GS in neurotypical Spanish adolescents and children.

**Table:** 

**What is Known:** *• To the best of our knowledge, instruments assessing the severity of gastrointestinal symptoms in children and adolescents are still too few*.	
**What is New:** *• The GSSS shows promising psychometric properties for assessing GS in neurotypical adolescents and children. The GSSS may help improve understanding of GS involvement in the gut-brain microbiota axis in children and adolescents*.

**Supplementary Information:**

The online version contains supplementary material available at 10.1007/s00431-024-05504-8.

## Introduction

Functional gastrointestinal disorders (FGIDs) are characterized by a number of chronic or recurrent gastrointestinal symptoms (GS) that are not explained by structural or biochemical abnormalities. In addition, such disorders significantly impinge upon quality of life in both the individual and their family. FGIDs are diagnosed and classified using standardized criteria, as outlined by the Rome Foundation. Rome IV criteria (2016) argues that such conditions should be considered in terms of their impact on gut–brain interaction, acknowledging the complex psycho-biosocial interaction inherent to their pathogenesis [1]. New Rome criteria reflect improved detection of FGIDs from that seen with the previous version [2].

Irritable bowel syndrome (IBS), functional dyspepsia and functional constipation are FGIDs with complex pathophysiology’s. Furthermore, a high prevalence of individuals with FGID-criteria meeting symptoms has been found in the general population, with FGIDs being more frequent in women [3]. Specifically, functional abdominal pain disorders are common disorders affecting between 3 and 16% of the neurotypical pediatric population [4]. GS prevalence has been found to be similar in non-clinical adolescent populations [2, 5]. Likewise, between 9.9 and 29% of neurotypical children and adolescents have been reported to suffer from FGIDs [5]. Specifically, this pertains to a prevalence of between 2 and 22.90% for acute diarrheal illness [6, 7], between 0.1 and 45.1% for irritable bowel syndrome, between 0.2 and 6.2% for cyclic vomiting, between 31.3 and 86.9% for functional constipation, 11.5% for dyspepsia, and between 2.4 and 55.1% for abdominal pain [2, 5, 8]. As a consequence, FGIDs affect quality of life [3, 9] places a strain on health resources [10].

Children and adolescents with and without neurodevelopmental disorders, such as Autism Spectrum Disorder (ASD), can frequently have gastro-intestinal problems (e.g., gastroesophageal reflux) associated with feeding problems. Such individuals may exhibit more restrictive dietary patterns (e.g., picky eaters) caused by sensitivity to certain foods. The avoidance of such foods may be associated with adverse circumstances (e.g., [11–14]). Cognitive rigidity and taste sensitivity appear to be significant predictors of selective or “picky” eating in children and adolescents, regardless of sex [14]. Further, associations have been found between anxiety, sensory reactivity and chronic abdominal pain [13, 15, 16]. Furthermore, research suggests a significant relationship between obsessive-compulsive symptoms of some mental disorders (e.g.: obsessive-compulsive disorder) and GS [17, 18]. In this way, studies indicate that there may be a relationship between GS, emotional instability and gut dysbiosis. Thus, the psychobiological symptoms discussed above can reveal the nature of the gut-microbiota- brain relationship [12, 19–24].

To the best of our knowledge, instruments assessing the severity of gastrointestinal symptoms in children and adolescents are still too few and far between. Some GS measurement instruments focus on measuring symptoms through information provided by caregivers (e.g. *Questionnaire on Pediatric Gastrointestinal Symptoms—Rome III [QPGS-RIII]*) [2] or are administered to adults to gather self-reported information (e.g. *Gastrointestinal Symptoms Severity Index [GISSI]*) [25]. A measure is, therefore, required which can be used to collect self-report data during the developmental period of childhood and adolescence. In this regard, adolescence is a transitional period during which a series of important psychophysiological changes occur. In this sense, there is a need to explore GS during this crucial life stage in order to develop a measure that can be administered during childhood, adolescence and adulthood [26]. Furthermore, recent studies highlight the need to develop new scales that address Rome IV criteria (e.g. [2, 3, 27]. This is due to the fact that the prevalence differs between Rome III and IV criteria due to changing symptomatology [2]. Therefore, there are a series of limitations to the scales that have been developed in the past to evaluate GS, among which is that the same scale has not been adapted for the developmental period (childhood, youth, adolescence and adulthood), nor is there a version for caregivers and professionals.

### Aims of the present study

Based on the aforementioned, the present research team developed a new instrument, the *Gastrointestinal Symptom Severity Scale* (GSSS). The following six objectives were outlined:

- Develop an instrument for assessing the severity of gastrointestinal symptoms, the GSSS.
- Examine the structural validity of the GSSS using sequential analysis comprising exploratory factor analysis (EFA) and confirmatory factor analysis (CFA) in a sample of neurotypical children and adolescents.
- Examine the internal consistency and test-retest reliability at 4 weeks of the GSSS in a sample of neurotypical children and adolescents.
- Examine measurement invariance of the GSSS, as a function of sex, in a sample of neurotypical children and adolescents.
- Examine validity of the GSSS for hypothesis testing by comparing outcomes with those produced by other related instruments in a sample of neurotypical children and adolescents.
- Provide descriptive data on GSSS in a sample of neurotypical children and adolescents.

## Methods

### Design

**Fig. 1:**
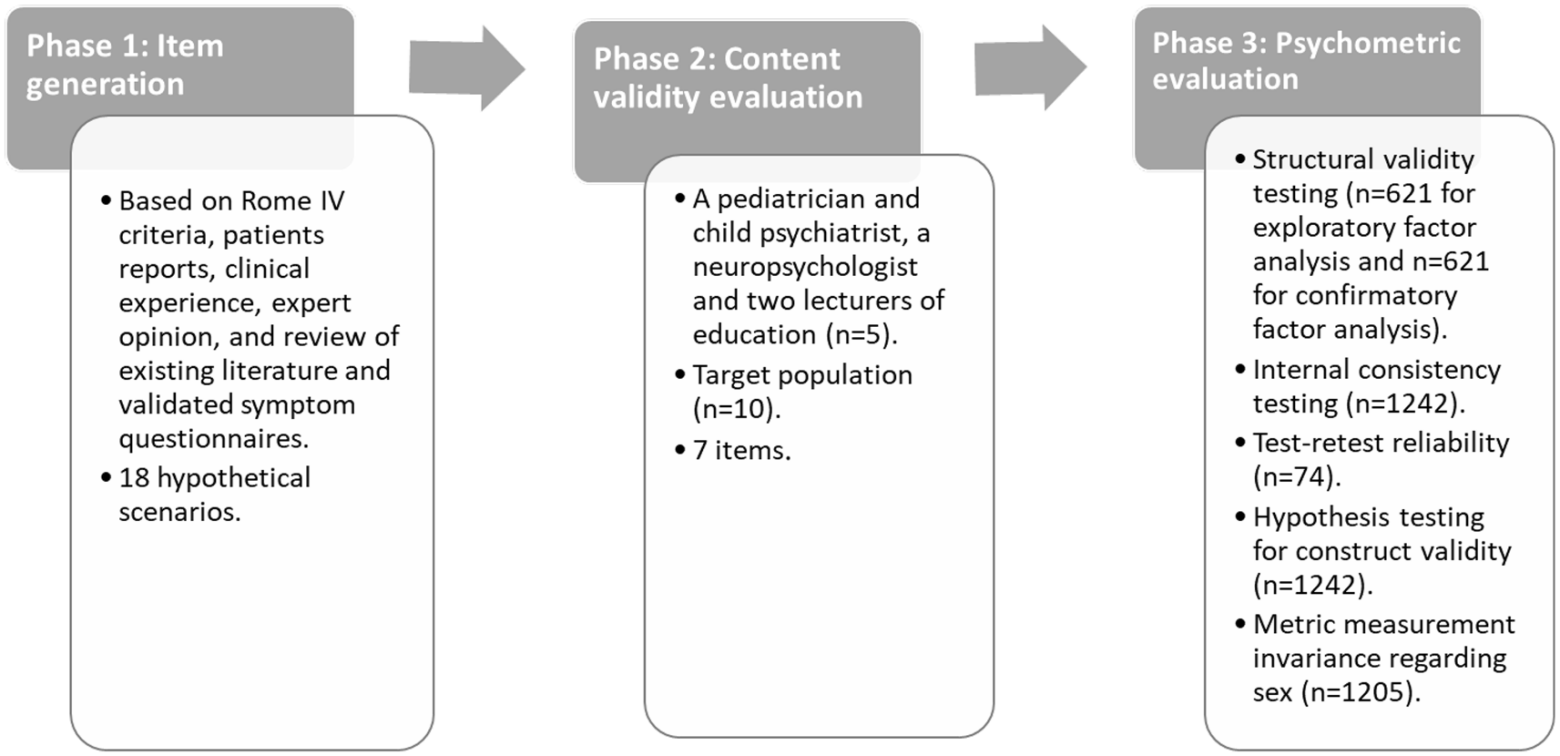
Phases of the GSSS development and validation study. Adapted from: Slavec and Drnovšek [ 30 ]

A web-based instrumental study was conducted to develop, validate, and examine the psychometric properties of the new instrument GSSS [28, 29] in a sample of neurotypical children and adolescents recruited in Spain. It consists of three phases adapted from the methodology outlined by Slavec and Drnovšek [30] (Fig. 1).

### Development and content validity of the new instrument

The purpose of developing the GSSS was to create a measure capable of providing information on the severity of gastro-intestinal symptoms in non-autistic and autistic children and adolescents. The main focus of the present study is on outcomes pertaining to the non-autistic group. Rome IV criteria were adhered to [1].

**Fig. 2:**
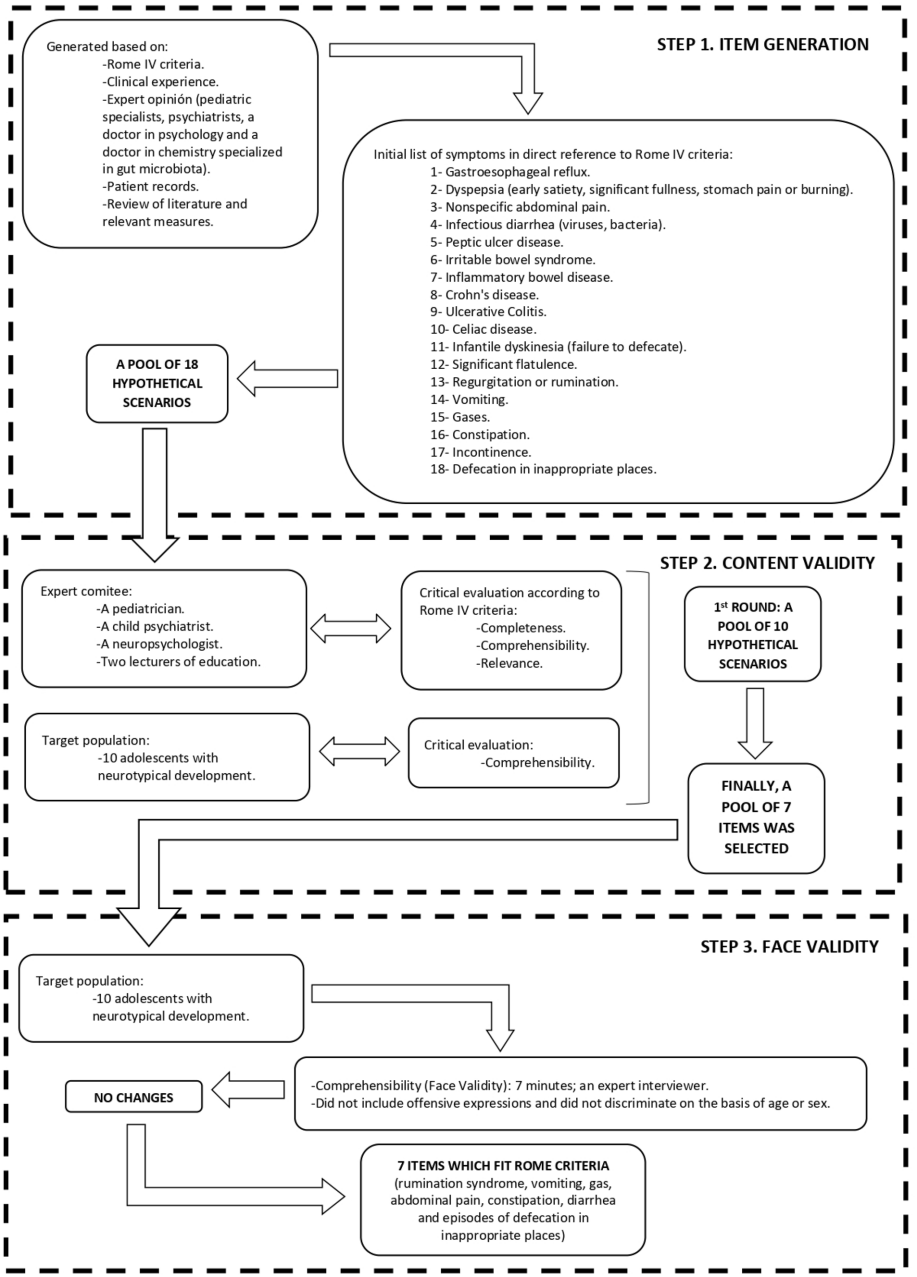
Development process of the GSSS

Figure 2 illustrates the instrument development process. The GSSS was developed by a multidisciplinary team (pediatric specialists, psychiatrists, a doctor in psychology and a doctor in chemistry specialized in gut microbiota) [31]. Initial items were generated based on Rome IV criteria, clinical experience, patient records, expert opinion, and review of existing literature and validated symptom questionnaires. An initial list of 18 symptoms was drawn in direct reference to Rome IV criteria [1].

Items were designed to garner information on the frequency with which a sensation was experienced, the degree of discomfort caused and the extent to which this sensation posed an issue to patients. A reported issue is deemed to be problematic in cases in which a sensation is either very intense or annoying, highly frequent, impedes the realization of daily living activities and/or leads to negative consequences for the individual or others.

In order to assess content validity and understanding of the items, the survey was administered to a pediatrician and child psychiatrist, a neuropsychologist and two lecturers of education. Critical evaluation focused on the content, completeness (as determined according to Rome IV criteria) and clarity of the survey. The revised instrument was pilot tested with 10 adolescents with neurotypical development, providing information of clarity of questions and administration time. The questionnaire was further revised based on feedback received from this pilot. From an initial list of 18 symptoms, a 10-item questionnaire was developed that assessed gastrointestinal symptoms. Items were submitted to evaluation and refinement processes as described above until a total of 7 items were retained which fit Rome criteria (rumination syndrome, vomiting, gas, abdominal pain, constipation, diarrhea and episodes of defecation in inappropriate places). Each item comprised a description of the condition with information being collected on the length of time, in weeks or months, that the gastro-intestinal symptom had been experienced. Finally, face validity was conducted with 10 adolescents with neurotypical development to ensure that all items were easy to understand, did not include offensive expressions and did not discriminate on the basis of age or sex. No difficulties in item understanding were observed.

### Participants

Participants were selected, via non-probabilistic convenience sampling, from nine schools in the regions of Alicante, Murcia and Andalusia (Spain). The selection process took place between March 2022 and May 2023. Eligibility criteria included: (1) aged 16 years and under, (2) children and adolescents with typical development, and (3) proficient in Spanish.

### Sample size

In consideration of the latest guidelines in the field of psychometrics developed by Ferrando et al. [32] and Lloret-Segura et al. [33], a sample size of at least 500 cases is recommended for performing EFA (*n* = 250) and CFA (*n* = 250), even under optimal conditions and with well-determined factors. The total study sample consisted of 1242 participants.

### Recruitment

Participants completed all study measures online using the web-based survey tool, LimeSurvey (LimeSurvey GmbH, Hamburg, Germany). At the beginning of each questionnaire, each participant was requested to enter a unique code generated by LimeSurvey and a valid email address to enable their later participation in the study. All codes and emails were analyzed to ensure that no participant responded multiple times. Consent to participate came from participant’s parents and/or caregivers in accordance with the Declaration of Helsinki. Appropriate instructions were provided on each instrument to enable completion of the web-based questionnaire. The total time required to complete all instruments was approximately 20 min. Participants completed all procedures in their classrooms. A researcher remained in the classroom throughout questionnaire administration to assist students who experienced difficulties.

### Measures

#### Clinical questionnaire on gastro-intestinal symptoms

This is an ad hoc questionnaire that was developed to identify gastro-intestinal disorders according to Rome IV criteria [1]. The tool comprises a series of questions regarding gastrointestinal disorders (e.g. diarrhea, abdominal pain, dyspepsia, gastroesophageal reflux, etc.) and family history.

#### Gastrointestinal Symptom Severity Scale (GSSS)

As discussed above, an instrument was elaborated based on Rome IV criteria [1] comprising 7 items regarding the main gastro-intestinal symptoms (constipation, diarrhea, average stool consistency, stool odor, abdominal pain, flatulence and gas). Scale items are rated on a four-point Likert scale ranging from 0 (none/nothing or this symptom does not occur) to 3 (very frequent and troublesome symptom). The severity of gastrointestinal symptoms was evaluated according to three criteria: (1) intensity or degree of discomfort, (2) it is very common, and 3) negatively affects other activities in daily life. The instrument has two versions, a web-based version for caregivers/professionals and a web-based version for children and adolescents. In the present study, the web-based version for children and adolescents was administered.

#### Pain and Sensitivity Reactivity Scale (PSRS)

The PSRS evaluates reactivity to pain and sensory reactivity through 50 items. It is composed of three dimensions: Pain, sensory hyporeactivity and sensory hyperreactivity. Items are rated on a four-point Likert scale ranging from 0 (behavior does not occur) to 3 (behavior occurs and is a severe problem). Sensory hyposensitivity and sensory hypersensitivity dimensions comprise tactile, olfactory, visual, gustatory and auditory items. The pain reactivity domain of the scale comprises seven items. The PSRS is based on theoretical postulates conceived by Miller et al. [34] pertaining to sensory modulation disorders, in which the proposed nosology for diagnosis separates such disorders according to three main patterns (hyper-response, hypo-response and sensory seeking). Two versions of the PSRS are available, with a version for caregivers/professionals and another self-report version for individuals themselves. The present study refers to the self-report version. Internal consistency of the overall scale and its subscales, examined according to Cronbach's alpha, has been shown to be good in a neurotypical Spanish population of young adults (PSRS-overall = .92; pain = .79; broad sensory hyporeactivity = .88; broad sensory hyperreactivity = .90) [35]. The caregiver version of the PSRS has also shown excellent internal consistency when administered to an Spanish ASD sample (pain = .83; broad sensory hyporeactivity = .90; broad sensory hyperreactivity = .93) [36].

#### Sensory Over-Responsivity Scales (SORS)

SORS assesses sensory hyperreactivity to auditory, tactile, visual, olfactory and taste stimuli. SORS was adapted from a measure used with a general community sample [37]. It consists of rating scales to measure distress and impairment in relation to both auditory and tactile over-reactivity [38]. Each scale on the SORS contains 4 questions that are rated on a 4-point scale, with overall scores ranging from 0 to 80. Overall subscale scores are calculated individually and range from 0 to 16, with higher scores indicating greater severity. Strong internal consistency, in accordance with Cronbach’s alpha, of SORS overall and its subscales has been shown in sample from the United States (SOR-overall = .93; SOR-hearing = .89; SOR-touch = .88; SOR-smell = .90; SOR-sight = .94; SOR-taste = .88) and in a sample from Spain (hearing = .89; touch = .86; smell = .91; sight = .90; taste = .86) [39].

#### Obsessive-Compulsive Inventory – Revised (OCI-R)

The OCI-R is an 18 item self-report questionnaire that assesses obsessive-compulsive symptom severity using a 5-point Likert scale ranging from 0 (not at all) to 4 (very much). The OCI-R comprises 6 factors that represent the following symptom domains: Checking, ordering, neutralizing, washing, obsessing and hoarding [40]. Each factor is composed of 3 items (possible range = 0–12). Overall, the measure has shown good internal consistency in samples from various countries, with Cronbach’s α ranging from .81 to .95 [41–43].

### Data analysis

The total number of observations (N = 1,242) was randomly divided into two samples, sample 1 (n = 621) and sample 2 (*n* = 621). All analytical procedures were performed using the free software R (version 6.3). The performance of the instrument was analyzed by calculating skewness, kurtosis, and floor and ceiling effects. Skewness and kurtosis coefficients greater than 1.5 or lower than - 1.5 indicate that the assumption of normality is violated [32, 33]. Floor and ceiling effects are considered present when more than 15% of participant responses correspond to extreme response categories (high end or low end) [29, 44]. According to criteria outlined by Rhemtulla et al. [45], data were considered to be ordinal. Instrument structure was evaluated using exploratory factor analysis (EFA) in data reported by sample 1. Adequacy of the EFA was evaluated using the Kaiser-Meyer-Olkin (KMO) test (acceptable values ≥ .70) [46], Bartlett’s test of sphericity (*p* < .05 being acceptable) [47] and the coefficient of determination (*R*^2^ close to 0 is acceptable) [32, 33]. In order to determine the number of factors comprised by the instrument, parallel analysis (PA) [32, 33, 48] and Cattell’s Scree Test (CTS) [49] were used. EFA was performed with the *“psych*” package [50] using the unweighted least squares (ULS) estimation method recommended for categorical variables when the normality assumption is violated, alongside Promax rotation [32, 33]. Item selection and retention criteria included: (a) saturation ≥ .40 and (b) elimination of Heywood cases (saturation ≥ 1) [51]. Subsequently, the structure obtained via EFA was evaluated through confirmatory factor analysis (CFA) using data collected from sample 2. For this, the “*Lavaan*” package [52] was used, employing the weighted least square means and variance adjusted (WLSMV) method, as recommended for ordinal variables [53]. Model fit was assessed using according to the comparative fit index (CFI), Tucker-Lewis index (TLI) and root mean square error of approximation (RMSEA), with CFI > .90, TLI > .90 and RMSEA < .06 being considered acceptable [29, 54]. Three statistical adjustments were proposed: (1) tau-equivalent, (2) congeneric and (3) correlated errors (modification indices > 35000). Models with Heywood cases, < 35000 correlated errors and negative variances were rejected [32, 33, 55]. Internal consistency was assessed for the overall sample by calculating the ordinal alpha coefficient, which provides a more precise estimate for categorical response scales. An α ≥ .70 indicates acceptable reliability [56, 57]. Test-retest reliability (*n* = 74) was evaluated after four weeks by calculating the interclass correlation coefficient (ICC). ICC values ≥ .60 are considered to be good [58]. Predictive power regarding hypothesis testing was evaluated by calculating product-moment correlations between relevant factors and items of the GSSS and other instruments measuring related but different constructs, in this case, the PSRS, SOR and OCI-R. Sufficient predictive power is shown through correlations of around 0.20–0.50 [29], which would confirm the hypothesis that the instrument measures what it was designed to measure. Measurement invariance as a function of sex, discarding all ‘other’ responses, was evaluated (*n* = 1,205) in accordance with the method outlined by Wu and Estabrook [59], which assesses four levels of invariance: (a) configural invariance; (b) metric invariance; (c) scalar invariance; (d) residual invariance. In this sense, ΔCFI and ΔRMSEA differences of ≤ .010 and ≤ .015, respectively, were considered to indicate insignificant measurement variance and show measurement invariance [60].

### Ethical considerations

The present study was approved by the Ethics Committee of the University of Alicante in Spain (reference: UA-2019-10-04. Approval Date: March 27, 2020).

## Results

### Socio-demographic and clinical characteristics of the sample

Sociodemographic characteristics of the sample are shown in Table 1. Of the 1242 participants evaluated, 85.3% were aged between 13 and 16 years, with 49.4% being female. A total of 94.7% of the sample was of Spanish nationality.

**Table 1:** Sociodemographic characteristics of the total sample

**Variables**	***n* (%)**
Years	
< years old	183 (14.7)
13 to 16 years old	1059 (85.3)
Sex	
Female	614 (49.4)
Male	591 (47.6)
Other	37 (3)
Country/region of origin	
Spain	1176 (94.7)
Rest of Europe	14 (1.1)
America	30 (2.4)
Africa	16 (1.3)
Asia	6 (0.5)

The presence of gastrointestinal disorders in the sample is detailed in the supplementary information (Supplementary Table S1). Specifically, in the present sample of children and adolescents with typical development, 13.8% were found to have infectious diarrhea, 12.6% abdominal pain, 5% dyspepsia and 2.6% gastroesophageal reflux.

### Psychometric assessment

Table 2 presents outcomes indicating the performance of instrument items. Floor effects, skewness and kurtosis were found meaning that data were considered to be ordinal.

**Table 2:** Item performance of the GSSS

**Items**	**Min**	**Max**	**M (SD)**	**Skewness**	**Kurtosis**	**F.E (%)**	**C.E (%)**
1. Regurgitation or rumination	0	3	.19 (.44)	2.51	7.32	1020 (82.1)	4 (.3)
2. Vomiting	0	3	.26 (.50)	1.96	4.15	941 (75.8)	5 (.4)
3. Gas	0	3	.49 (.68)	1.39	1.89	735 (59.2)	23 (1.9)
4. Abdominal pain	0	3	.37 (.66)	1.93	3.57	881 (70.9)	23 (1.9)
5. Constipation	0	3	.30 (.60)	2.29	5.62	941 (75.8)	19 (1.5)
6. Diarrhea	0	3	.25 (.50)	2.16	5.40	957 (77.1)	7 (.6)
7. Defecation in inappropriate place	0	3	.08 (.32)	4.64	24.74	1149 (92.5)	2 (.2)

*FE* floor effect, *CE* ceiling effect, *M* mean *SD* standard deviation *Min* minimum, *Max* maximum

#### Exploratory factor analysis

**Fig. 3:**
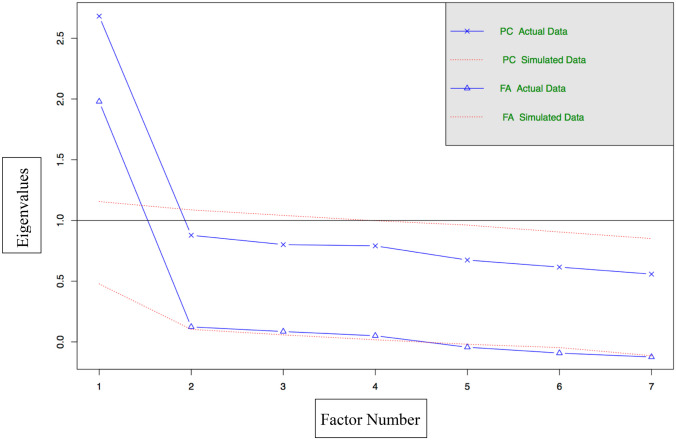
Factor extraction plot of the GSSS

Factor extraction was carried out using PA and CST (Fig. 3). Subsequently, EFA was carried out using the initial set of 7 items. This EFA produced a KMO ≥ .70, Bartlett *p* value < .05 and a coefficient of determination that was close to zero. In accordance with pre-determined criteria, no item was eliminated based on these outcomes. Table 3 presents factor loadings pertaining to all items. The GSSS explains a moderate percentage of overall variance, 39.92%.

**Table 3:** Results of exploratory factor analysis of the GSSS

**Items**	**Factor**
	**1**
1. Regurgitation or rumination	.50
2. Vomiting	.46
3. Gas	.54
4. Abdominal pain	.66
5. Constipation	.59
6. Diarrhea	.60
7. Defecation in inappropriate place	.42
**Explained variance %**	39.92

#### Confirmatory factor analysis

Outcomes of the adjusted CFA performed to meet predefined criteria are presented in Table 4.

**Table 4:** Results of confirmatory factor analysis of the GSSS

**Models**	***χ*2**	***df***	**RMSEA** **(90% CI)**	**CFI**	**TLI**
TM	96.235	20	.064 (.048–.081)	.848	.840
CM	16.382	14	.017 (.000–.044)	.983	.975
CE**	-	-	-	-	-

*RMSEA* Root Mean Squared Error of Approximation, *CFI* Comparative Fit Index, *TLI* Tucker-Lewis Index, *CI* Confident Interval, *TM* Tau-Equivalent Model, *CM* Congeneric Model, *CE* Correlated Error Model

**Models rejected on the basis of previously agreed criteria

**Fig. 4:**
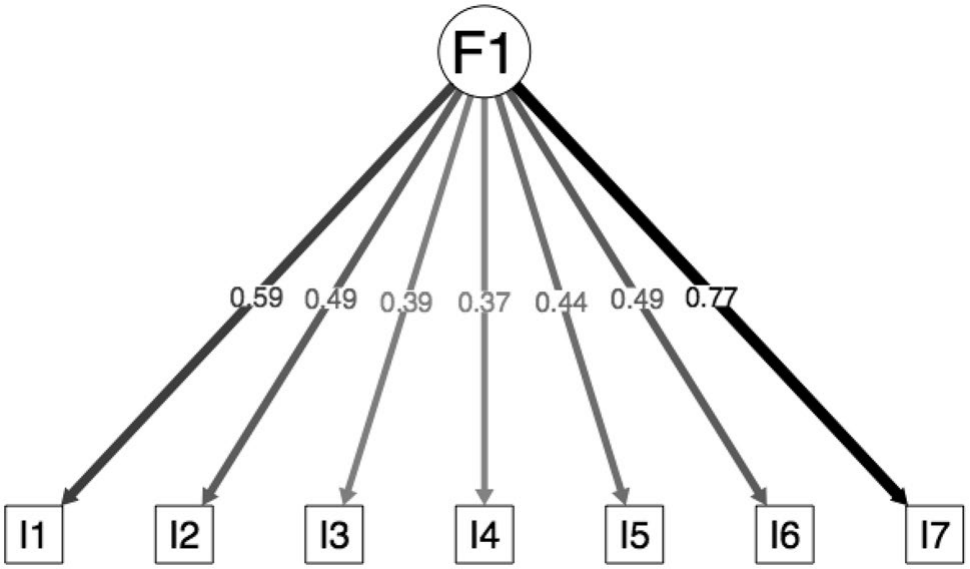
Factor loadings of the confirmatory factor analysis for the tau equivalent model

**Fig. 5:**
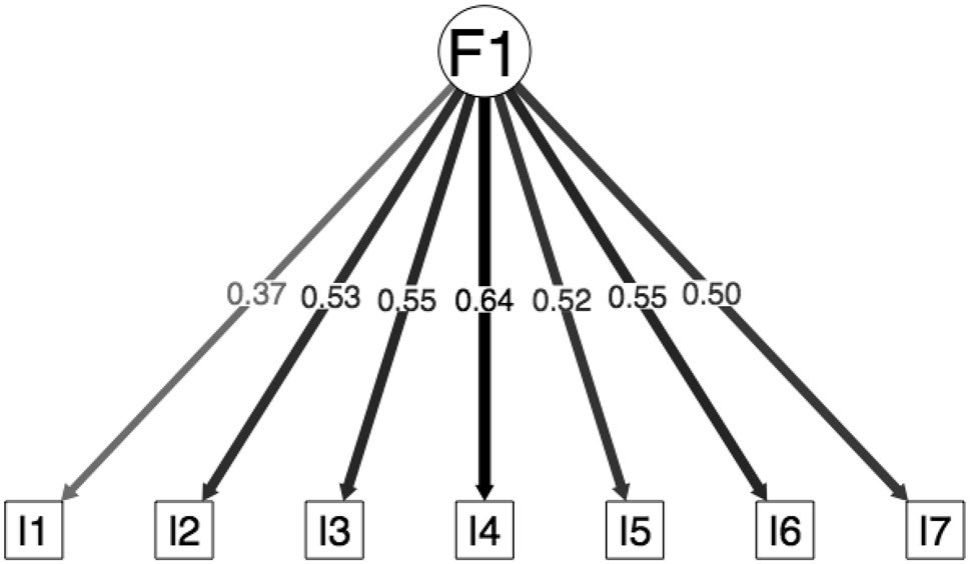
Factor loadings of the confirmatory factor analysis for the congeneric model

The tau-equivalent model (Fig. 4) presents marginal fit. The congeneric model supported following EFA presents excellent fit, with factor loadings ranging between .37 and .64 (Fig. 5).

#### Internal consistency and reliability

The GSSS shows an internal consistency for the total scale of .73 measured with ordinal alpha. The GSSS shows a test-retest reliability at 4 weeks of .71 (CI: .587-.812).

#### Measurement invariance

Measurement invariance outcomes are presented in Table 5. Outcomes indicate that metric measurement invariance regarding sex can be assumed, since none of the comparisons revealed a change in fit outside of the range of ΔCFI ≤ .010 or ΔRMSEA ≤ .015.

**Table 5:** Measurement Invariance as a function of sex

**Model**	**X**^**2**^	**gl**	**CFI**	**ΔCFI**	**RMSEA** **(90% CI)**	**ΔRMSEA**
Configurational	31.655	28	.986	-	.015 (.000–.036)	-
Metric	**38.202**	**34**	**.984**	**-.002**	**.014** **(.000–.034)**	**-.001**
Scalar	95.238	40	.790	-.194	.048 (.036–.061)	.034
Strict	115.078	47	.741	-.049	.049 (.038–.061)	.001

*RMSEA* Root Mean Squared Error of Approximation, *CFI* Comparative Fit Index, *TLI* Tucker-Lewis Index, *CI* Confident Interval

#### Hypotheses testing for construct validity

Product-moment correlations between factor scores reported for the GSSS, and PSRS, SOR and OCI-R scores are presented in Table 6. Overall, PSRS, SOR and OCI-R scores correlate positively with GSSS scores (*r* = .278 to .924; *p* < .01), with correlations being in the expected direction and of the expected magnitude.

**Table 6:** Hypothesis testing for construct validity

**SSGS**		
**PSRS**	Pain	.36**
	Total Hypo	.47**
	Hypo- Tactile	.99**
	Hypo-Olfactory	.39**
	Hypo-Visual	.35**
	Hypo-Taste	.35**
	Hypo-Auditory	.35**
	Total Hyper	.39**
	Hyper-Tactile	.34**
	Hyper-Olfactory	.31**
	Hyper-Visual	.28**
	Hyper-Taste	.30**
	Hyper-Auditory	.27**
**SOR**	Touch	.32**
	Smell	.28**
	Sight	.30**
	Taste	.27**
	Hearing	.31**
**OCI-R**	Hoarding	.31**
	Checking	.30**
	Ordering	.29**
	Neutralizing	.28**
	Washing	.28**
	Obsessing	.35**

*GSSS* Gastrointestinal Symptom Severity Scale, *PSRS* Pain and Sensitivity Reactivity Scale, *OCI-R* Obsessive Compulsive Inventory – Revised, *SOR* Sensory Over-Responsivity Scale, *Total Hypo* Total Sensory Hyporeactivity, *Total Hyper* Total Sensory Hyperreactivity, ** = *p* < 0.01

#### GSSS Descriptive Statistics

Supplementary information Table S2 presents mean and percentage GSSS outcomes for the overall sample and according to sex. No significant differences are observed between females and males.

## Discussion

### Main findings

The study aimed to evaluate the psychometric properties of the new GSSS instrument in a Spanish sample of neurotypical children and adolescents. The end result was a 7-item questionnaire with a 4-point Likert scale which measures the severity of GS (Supplementary information S3). The study findings show that the GSSS has acceptable and promising psychometric properties.

The prevalence of GS in different countries, cultures and life stages is of great interest to the scientific community and in professional practice. Present outcomes regarding the prevalence of FDIGs in neurotypical children and adolescents are highly similar to those reported in other countries [2, 5–8].

GS can be present in the child and adolescent population and in adults, emerging in both clinical and non-clinical populations (e.g., [2, 4, 5]). Questionnaires such as the GSSS can be helpful in analyzing the severity of GS at important life stages in clinical populations [13]. In this way, the trajectory of gastro-intestinal development can be identified.

A previous study administering the GSSS to a sample of individuals with ASD showed it to have a single-factor structure [61]. Findings obtained in the present study confirm the presence of a single-factor structure, which was corroborated through the use of sequential analysis in the form of EFA and CFA. Excellent fit indices, in line with that recommended in existing literature, were obtained for the congeneric model using CFA [29, 54]. Acceptable internal consistency of the GSSS was also found (≥ .70), with this being in line with that reported by previously conducted research [56, 57]. In addition, test-retest reliability was good (≥ .60) [29, 58]. These findings are similar to those reported by other validation studies using instruments with similar characteristics to the GSSS and conducted with adult populations (e.g., [25]).

In terms of measurement invariance, the GSSS exhibited metric measurement invariance. This is a great advantage as it allows for meaningful comparisons between sex, ensuring that the same construct is measured consistently across groups. This not only facilitates valid comparisons, but, also, allows researchers to draw accurate conclusions regarding differences or similarities between males and females [59].

In relation to predictive power, the initially proposed hypothesis is confirmed. Significant correlations, ranging from moderate to strong, were observed between the GSSS and the dimensions of hyporeactivity and hyperreactivity included on the PSRS. Additionally, significant positive correlations were identified between GSSS, SORS and the OCI-R. These findings align with previous research indicating a relationship between sensory reactivity, pain and GS [13, 15, 16]. Such associative patterns are evident, not only overall, but, more notably, in relation to tactile stimuli. An explanation for these findings may be found in the functioning of the numerous mechanosensory circuits distributed throughout the GI tract. These circuits rely on a range of proposed specialized and non-specialized mechanosensory cells that include epithelial enterochromaffin cells, sensory neurons, glia, interstitial cells of Cajal and smooth muscle cells. The neuro-epithelial mechanosensory circuit in the gut and the light touch circuit in the skin have many similar characteristics, including their implication in gastro-intestinal health [62].

In relation to the descriptive statistics of the GSSS, we provide percentiles of the instrument within our context to identify individuals at risk of developing and/or and/or suffering from severe GS. Based on these reference points, it is possible to identify outlier values, which would suggest the need for evaluation by a medical professional [63].

## Strengths and limitations

The main strengths of the present study are the high methodological and psychometric standards applied to the validation of the GSSS. Furthermore, confirmation of measurement invariance represents a state-of-the-art approach with strong practical implications regarding the interpretation of group differences. Despite these strengths, it is crucial to mention that while percentiles have been provided to identify individuals at risk of developing and/or suffering from severe GS, these should be considered with caution. Future studies would benefit from calculating the sensitivity and specificity of the instrument using AUC-ROC curves, taking into consideration a clinical gold standard. This approach would enhance the instrument’s applicability. Also, as seen in previous studies, one of the limitations of the present study is that the self-report version of the instrument was applied [25]. Although a caregiver version of the GSSS is available, it was considered important to gather psychometric data on the self-report version as a crucial first step towards determining psychometric robustness of the instrument. In the future, it will be possible to improve inter-rater reliability. Additionally, the GSSS is a web-based instrument created for a specific population. New validation and adaptation procedures are needed to adapt the instrument for use in new contexts and languages.

## Conclusion

In conclusion, the GSSS enables brief assessment of the severity of GS inneurotypical children and adolescents. Its psychometric properties suggest that it is suitable for use with children ranging between 13 and 16 years in Spain using a web-based survey. The GSSS represents a potentially hugely useful tool for medical professionals, diagnosis of FGIDs and analysis of the gut-microbiota-brain axis. It represents a new contribution to the evaluation of GS in children and adolescents through self-report questionnaires.

### Supplementary Information

Below is the link to the electronic supplementary material.

Supplementary file1 (DOCX 25 KB)

## Data Availability

No datasets were generated or analysed during the current study.

## References

[1] Drossman DA, Hasler WL (2016). Rome IV—Functional GI disorders: disorders of gut-brain interaction. Gastroenterology.

[2] Baaleman DF, Velasco-Benítez CA, Méndez-Guzmán LM, Benninga MA, Saps M (2021). Functional gastrointestinal disorders in children: agreement between Rome III and Rome IV diagnoses. Eur J Pediatr.

[3] Sperber AD, Bangdiwala SI, Drossman DA, Ghoshal UC, Simren M, Tack J, Whitehead WE, Dumitrascu DL, Fang X, Fukudo S et al (2021) Worldwide prevalence and burden of functional gastrointestinal disorders, results of Rome Foundation Global Study. Gastroenterology 160:99–114.e113. 10.1053/j.gastro.2020.04.014 10.1053/j.gastro.2020.04.014 32294476

[4] Thapar N, Benninga MA, Crowell MD, Di Lorenzo C, Mack I, Nurko S, Saps M, Shulman RJ, Szajewska H, van Tilburg MAL (2020). Paediatric functional abdominal pain disorders. Nat Rev Dis Primers.

[5] Boronat AC, Ferreira-Maia AP, Matijasevich A, Wang Y-P (2017). Epidemiology of functional gastrointestinal disorders in children and adolescents: a systematic review. World J Gastroenterol.

[6] Badawi MM, SalahEldin MA, Idris AB, Idris EB, Mohamed SG (2024). Diarrheal diseases prevalence among children of Sudan and socio cultural risks related; systematic review and meta analysis. BMC Infect Dis.

[7] Cui P, Li J, Liu N, Duan ZJ (2018). Incidence of acute diarrheal illness in Chinese communities: a meta-analysis. BMC gastroenterol.

[8] Vesterling C, Schütz-Wilke J, Bäker N, Bolz T, Eilts J, Koglin U, Rademacher A, Goagoses N (2023). Epidemiology of somatoform symptoms and disorders in childhood and adolescence: a systematic review and meta-analysis. Health Soc Care Community.

[9] Black CJ, Drossman DA, Talley NJ, Ruddy J, Ford AC (2020). Functional gastrointestinal disorders: advances in understanding and management. The Lancet.

[10] Aziz I, Palsson OS, Törnblom H, Sperber AD, Whitehead WE, Simrén M (2018). The prevalence and impact of overlapping Rome IV-diagnosed functional gastrointestinal disorders on somatization, quality of life, and healthcare utilization: a cross-sectional general population study in three countries. American College of Gastroenterology.

[11] Marco E, Janette S, Raffaele N, Fadda R, Francesca F, Eleonora N, Giovanni V, Stefano V (2019). Sensory processing, gastrointestinal symptoms and parental feeding practices in the explanation of food selectivity: clustering children with and without autism. Int j autism relat disabil.

[12] Martínez-González AE, Andreo-Martínez P (2019). The role of gut microbiota in gastrointestinal symptoms of children with ASD. Medicina.

[13] Martínez-González AE, Rodríguez-Jiménez T, Piqueras JA, Infante-Cañete L, Hidalgo Berutich S, Andreo-Martínez P, Ordóñez-Rubio T, Belmonte Lillo VM, Cubi MA, Navarro-Soria I (2024). Cross-disorder comparison of sensory reactivity, pain, gastro-intestinal symptoms and obsessive-compulsive symptoms in adolescents and young adults with autism and other neurodevelopmental disorders. Int J Dev Disabil.

[14] Zickgraf HF, Richard E, Zucker NL, Wallace GL (2022). Rigidity and sensory sensitivity: independent contributions to selective eating in children, adolescents, and young adults. J Clin Child Adolesc Psychol.

[15] Mazurek MO, Keefer A, Shui A, Vasa RA (2014). One-year course and predictors of abdominal pain in children with autism spectrum disorders: the role of anxiety and sensory over-responsivity. Res Autism Spectr Disord.

[16] Mazurek MO, Vasa RA, Kalb LG, Kanne SM, Rosenberg D, Keefer A, Murray DS, Freedman B, Lowery LA (2013). Anxiety, sensory over-responsivity, and gastrointestinal problems in children with autism spectrum disorders. J Abnorm Child Psychol.

[17] Marazziti D, Buccianelli B, Palermo S, Parra E, Arone A, Beatino MF, Massa L, Carpita B, Barberi FM, Mucci F (2021). The microbiota/microbiome and the gut–brain axis: how much do they matter in psychiatry?. Life.

[18] Turna J, Grosman Kaplan K, Patterson B, Bercik P, Anglin R, Soreni N, Van Ameringen M (2019). Higher prevalence of irritable bowel syndrome and greater gastrointestinal symptoms in obsessive-compulsive disorder. J Psychiatr Res.

[19] Martínez-González AE, Andreo-Martínez P (2020). Prebiotics, probiotics and fecal microbiota transplantation in autism: a systematic review. Rev Psiquiatr Salud Ment.

[20] Martínez-González AE, Andreo-Martínez P (2020) Implications of gut microbiota and gastrointestinal symptoms in autism. In Advances in Health and Disease, Duncan, L.T., Ed.; NOVA Science Publishers: USA, Volume 29,16–21.

[21] Simpson CA, Diaz-Arteche C, Eliby D, Schwartz OS, Simmons JG, Cowan CSM (2021). The gut microbiota in anxiety and depression – a systematic review. Clin Psychol Rev.

[22] Andreo-Martínez P, García-Martínez N, Sánchez-Samper EP, Martínez-González AE (2019). An approach to gut microbiota profile in children with autism spectrum disorder. Environ Microbiol Rep.

[23] Andreo-Martínez P, García-Martínez N, Sánchez-Samper EP, Quesada-Medina J, MacFabe D (2018). Metabolites of the gut microbiota involved in the autism spectrum disorder. Rev. Dis. Cli. Neuro..

[24] Andreo-Martínez P, Rubio-Aparicio M, Sánchez-Meca J, Veas A, Martínez-González AE (2022). A Meta-analysis of gut microbiota in children with autism. J Autism Dev Disord.

[25] Crowell MD, Umar SB, Lacy BE, Jones MP, DiBaise JK, Talley NJ (2015). Multi-Dimensional Gastrointestinal Symptom Severity Index: validation of a brief GI symptom assessment tool. Dig Dis Sci.

[26] Vriesman MH, Koppen IJN, Camilleri M, Di Lorenzo C, Benninga MA (2020). Management of functional constipation in children and adults. Nat Rev Gastroenterol Hepatol.

[27] Vernon-Roberts A, Alexander I, Day AS (2021). Systematic review of pediatric functional gastrointestinal disorders (Rome IV criteria). J Clin Med.

[28] Carretero-Dios H, Pérez C (2005) Normas para el desarrollo y revisión de estudios instrumentales. Int J Clin Health Psychol 5, 521–551. https://www.redalyc.org/pdf/337/33705307.pdf

[29] Prinsen CAC, Mokkink LB, Bouter LM, Alonso J, Patrick DL, de Vet HCW, Terwee CB (2018). COSMIN guideline for systematic reviews of patient-reported outcome measures. Qual Life Res.

[30] Slavec A, Drnovšek M (2012). A perspective on scale development in entrepreneurship research. Econ Bus Rev.

[31] Lynn MR (1986). Determination and quantification of content validity. Nurs Res.

[32] Ferrando PJ, Lorenzo-Seva U, Hernández-Dorado A, Muñiz J (2022). Decalogue for the factor analysis of test items. Psicothema.

[33] Lloret-Segura S, Ferreres-Traver A, Hernández-Baeza A, El T-M (2014). análisis factorial exploratorio de los ítems: una guía práctica, revisada y actualizada. Anales de psicología/annals of psychology.

[34] Miller LJ, Anzalone ME, Lane SJ, Cermak SA, Osten ET (2007). Concept evolution in sensory integration: a proposed nosology for diagnosis. Am J Occup Ther.

[35] Martínez-González AE, Montoro-Pérez N, Wallace A, Pérez-Sánchez S, Piqueras JA, Infante-Cañete L, Hidalgo-Berutich S, Rodríguez-Jiménez T, Andreo-Martínez P. (2024). Psychometric Properties of the Gastrointestinal Symptom Severity Scale in a Sample of adolescents and young adults. J Clin Med 13(6):1662. 10.3390/jcm13061662 10.3390/jcm13061662 PMC10971376 38541887

[36] Martínez-González AE, Cervin M, Piqueras JA, Infante-Cañete L, Pérez-Sánchez S (2023). Psychometric properties of the Pain Reactivity and Sensitivity Scale in a diverse sample of autistic people.

[37] Taylor S, Conelea CA, McKay D, Crowe KB, Abramowitz JS (2014). Sensory intolerance: latent structure and psychopathologic correlates. Compr Psychiatry.

[38] Falkenstein MJ, Conelea CA, Garner LE, Haaga DAF (2018). Sensory over-responsivity in trichotillomania (hair-pulling disorder). Psychiatry Res.

[39] Moreno-Amador B, Cervin M, Martínez-González AE, Piqueras JA (2023). Sensory overresponsivity and symptoms across the obsessive-compulsive spectrum: web-based longitudinal observational study. J Med Internet Res.

[40] Foa EB, Huppert JD, Leiberg S, Langner R, Kichic R, Hajcak G, Salkovskis PM (2002). The Obsessive-Compulsive Inventory: development and validation of a short version. Psychol Assess.

[41] Hon KS, Siu BW, Cheng C, Wong WC, Foa EB (2019). Validation of the Chinese version of obsessive-compulsive inventory-revised. East Asian Arch Psychiatry.

[42] Piqueras Rodríguez JA, Martínez González AE, Hidalgo Montesinos MD, Fullana Rivas MA, Mataix Cols D, Rosa Alcázar AI (2009). Psychometric properties of the Obsessive Compulsive Inventory-revised in a non-clinical sample of late adolescents. Psicol Conductual.

[43] Martínez-González AE, Piqueras JA, Marzo JC (2011). Validación del inventario de obsesiones y compulsiones revisado (OCI-R) para su uso en población adolescente española. Anales De Psicología/Annals of Psychology.

[44] Lim JS, Lim MY, Choi Y, Ko G (2017). Modeling environmental risk factors of autism in mice induces IBD-related gut microbial dysbiosis and hyperserotonemia. Mol Brain.

[45] Rhemtulla M, Brosseau-Liard PÉ, Savalei V (2012). When can categorical variables be treated as continuous? A comparison of robust continuous and categorical SEM estimation methods under suboptimal conditions. Psychol Methods.

[46] Kaiser HF (1970). A second generation little jiffy. Psychometrika.

[47] Bartlett MS (1950). Tests of significance in factor analysis. Br J Stat Psychol.

[48] Hayton JC, Allen DG, Scarpello V (2004). Factor retention decisions in exploratory factor analysis: a tutorial on parallel analysis. Organ Res Methods.

[49] Cattell RB (1966). The scree test for the number of factors. Multivariate Behav Res.

[50] Revelle W (2016). How to: use the psych package for factor analysis and data reduction.

[51] Yong AG, Pearce S (2013). A beginner’s guide to factor analysis: focusing on exploratory factor analysis. TQMP.

[52] Rosseel Y, Oberski D, Byrnes J, Vanbrabant L, Savalei V, Merkle E, Hallquist M, Rhemtulla M, Katsikatsou M, Barendse M (2017) Package ‘lavaan’. Retrieved June, 17

[53] Beauducel A, Herzberg PY (2006). On the performance of maximum likelihood versus means and variance adjusted weighted least squares estimation in CFA. Struct Equ Modeling.

[54] Hu Lt, Bentler PM (1999). Cutoff criteria for fit indexes in covariance structure analysis: conventional criteria versus new alternatives. Struct Equ Modeling.

[55] Brown TA (2015) Confirmatory factor analysis for applied research. 2nd ed. The Guilford Press: New York, US, 462

[56] Gadermann AM, Guhn M, Zumbo BD (2019). Estimating ordinal reliability for Likert-type and ordinal item response data: a conceptual, empirical, and practical guide. Pract Assess Res Eval.

[57] Zumbo BD, Kroc E (2019). A measurement is a choice and Stevens’ Scales of Measurement do not help make it: a response to Chalmers. Educ Psychol Meas.

[58] Martínez Pérez JA, Pérez Martin PS (2023). Coeficiente de correlación intraclase. Medicina de Familia. SEMERGEN.

[59] Wu H, Estabrook R (2016). Identification of confirmatory factor analysis models of different levels of invariance for ordered categorical outcomes. Psychometrika.

[60] Chen FF (2007). Sensitivity of goodness of fit indexes to lack of measurement invariance. Struct Equ Modeling.

[61] Martínez-González AE, Cervin M, Pérez-Sánchez S (2024). Prevalence and correlates of gastrointestinal symptoms in people with autism: applying a new measure based on the Rome IV criteria.

[62] Treichel AJ, Farrugia G, Beyder A (2018). The touchy business of gastrointestinal (GI) mechanosensitivity. Brain Res.

[63] Schoonjans F, De Bacquer D, Schmid P (2011). Estimation of population percentiles. Epidemiology.

