# Supplementary material for: Development and validation of the Gastrointestinal Symptom Severity Scale in Spanish children and adolescents
Source: Eur J Pediatr. 2024 Mar 25;183(6):2703–15. doi: 10.1007/s00431-024-05504-8 (PMC11098890; doi:10.1007/s00431-024-05504-8)
Supplement: Supplementary file 1 — Supplementary file1 (DOCX 25 KB) [file 431_2024_5504_MOESM1_ESM.docx]

**Supplementary Table S1.**

*Presence of gastrointestinal disorders in the total sample.*

| **Gastrointestinal disorders** | ***n* (%)** |
| --- | --- |
| Infectious diarrhea | 172 (13.8) |
| Nonspecific abdominal pain | 157 (12.6) |
| Dyspepsia | 62 (5.0) |
| Gastroesophageal reflux | 32 (2.6) |
| Significant Flatulence | 24 (1.9) |
| Irritable Bowel Syndrome | 11 (.9) |
| Infantile Dyschezia | 11 (.9) |
| Inflammatory Bowel Disease | 6 (.5) |
| Celiac Disease | 11 (.9) |
| Ulcerative Colitis | 2 (.2) |
| Peptic Ulcer Disease | 4 (.3) |
| Crohn's disease | 2 (.2) |

**Supplementary Table S2.**

*Percentiles of the SSGS.*

|  |  |  | **M (SD)** | **Percentile** | **Severity level of gastrointestinal symptoms** | **Scale direct score** |
| --- | --- | --- | --- | --- | --- | --- |
|  |  |  |  | 29 | Very Low | 0 |
|  |  |  |  | 49 | Low | 1 |
|  | **Total SSGS** |  | 1.94 (2.36) | 69 | Medium-low | 2 |
|  |  |  |  | 79 | Medium-High | 3 |
|  |  |  |  | 89 | High | 5 |
|  |  |  |  | 99 | Very High | 11 |
|  |  |  |  |  |  |  |
|  |  |  |  | 29 | Very Low | 0 |
|  |  |  |  | 49 | Low | 1 |
|  | **Females** |  | 1.96 (2.19) | 69 | Medium-Low | 2 |
|  |  |  |  | 79 | Medium-High | 3 |
|  |  |  |  | 89 | High | 4 |
|  |  |  |  | 99 | Very High | 10 |
|  |  |  |  |  |  |  |
|  |  |  |  | 29 | Very Low | 0 |
|  | **Males** |  |  | 49 | Low | 1 |
|  |  |  | 1.88 (2.44) | 69 | Medium-Low | 2 |
|  |  |  |  | 79 | Medium-High | 3 |
|  |  |  |  | 89 | High | 5 |
|  |  |  |  | 99 | Very High | 11 |
|  |  |  |  |  |  |  |
|  |  |  |  | 29 | Very Low | 0 |
|  |  |  |  | 49 | Low | 2 |
|  | **Others** |  | 2.59 (3.51) | 69 | Medium-Low | 3 |
|  |  |  |  | 79 | Medium-High | 4 |
|  |  |  |  | 89 | High | 8 |
|  |  |  |  | 99 | Very High | 9 |
| **Contrast Statistics (Effect Size)** | | | | | ***p value*** | **Hedges’ g** |
| Total SSGS vs. Females | | | |  | > .05 | .0086 |
| Total SSGS vs Males | | | |  | > .05 | .0251 |
| Total SSGS vs. Others | | | |  | < .05 | .2708 |
| Females vs. Males | | | |  | > .05 | .0345 |
| Females vs. Others | | | |  | < .05 | .2759 |
| Males vs. Others | | | |  | < .05 | .2824 |

*Notes.* M=Mean; SD= Standard Deviation.

.

**Supplementary Material S3**

*Gastrointestinal Symptom Severity Scale (GSSS)*

Gastrointestinal symptoms (e.g., diarrhea, constipation) can be due to multiple causes (infections, etc.) and are experienced in relation to a certain frequency (temporary, recurring, etc.). Please read each of the gastro-intestinal symptoms below. Next, select the number that best describes the extent to which this happens to you and is a problem for you. When we talk about a problem, we mean that:

1) it is very intense, it is highly bothersome,

2) it is very common,

3) it negatively affects other activities in your life, causes you to avoid certain activities, people and/or places, and/or generates negative consequences for you and/or the people around you.

Make evaluations based on your most recent experiences.

| 0 = None/nothing (this symptom does not occur)  1 = Sometimes, but not a real problem  2 = Often and it is a real problem  3 = Very common and problematic |
| --- |

| 1. I experience REGURGITATION or RUMINATION  (chewing food that has already been digested and returned from the stomach to the mouth)  Note: This problem does not appear during sleep and does not cause respiratory distress.  Duration: For at least multiple consecutive hours or days. | 0 | 1 | 2 | 3 |
| --- | --- | --- | --- | --- |
| 2. I have difficulties with VOMITING  Duration: For at least multiple consecutive hours or days. | 0 | 1 | 2 | 3 |
| 3. I have GASES  (e.g., repetitive belching or increased flatulence)  Duration: For at least two consecutive months. | 0 | 1 | 2 | 3 |
| 4. I have ABDOMINAL PAIN  Note: You should observe a change in the frequency and shape of your stools (e.g., harder stools, feeling very full after meals, etc.).  Duration: Four days a month for at least two consecutive months. | 0 | 1 | 2 | 3 |
| 5. I have CONSTIPATION  Two or fewer bowel movements or feces a week. In addition, there may be a tendency to retain significant stool, the stool may be hard or very large and defecation may be painful.  Duration: One episode of incontinence per week. | 0 | 1 | 2 | 3 |
| 6. I have DIARRHEA  (loose or watery stools or stools that are painless)  Duration: For more than a month, without a justifying cause. | 0 | 1 | 2 | 3 |
| 7. I have EPISODES OF DEFECATION IN INAPPROPRIATE PLACES  Note: These episodes occur when you have sphincter control but find it difficult to retain stool.  Duration: Episodes have occurred for at least a month. | 0 | 1 | 2 | 3 |
